# Interaction Between Glycoside Hydrolase FsGH28c from *Fusarium solani* and PnPUB35 Confers Resistance in *Piper nigrum*

**DOI:** 10.3390/ijms26094189

**Published:** 2025-04-28

**Authors:** Shichao Liu, Tianci Xing, Ruibing Liu, Shengfeng Gao, Jianfeng Yang, Tian Tian, Chong Zhang, Shiwei Sun, Chenchen Zhao

**Affiliations:** 1 Spice and Beverage Research Institute, Chinese Academy of Tropical Agricultural Sciences, Wanning 571533, China; liushichao29@163.com (S.L.); liuliu_0608@163.com (R.L.); gsfkl@163.com (S.G.); xlzwyyjf@126.com (J.Y.); 18336506658@163.com (T.T.); 2 Key Laboratory of Genetic Improvement and Quality Regulation for Tropical Spice and Beverage Crops of Hainan Province, Wanning 571533, China; 3 College of Plant Protection, Shenyang Agricultural University, Shenyang 110866, China; 18339805261@163.com (T.X.); zhangchong0816@syau.edu.cn (C.Z.); 4 College of Plant Protection, Henan Agricultural University, Zhengzhou 450002, China

**Keywords:** glycoside hydrolase, *Fusarium solani*, cell death

## Abstract

Pathogens deploy various molecular mechanisms to overcome host defenses, among which glycoside hydrolases (GHs) play a critical role as virulence factors. Understanding the functional roles of these enzymes is essential for uncovering pathogen–host interactions and developing strategies for disease management. *Fusarium* wilt has occurred in the main *Piper nigrum* cultivation regions, which seriously affects the yield and quality of *P. nigrum*. Here, we identified and characterized FsGH28c, a GH28 family member in *Fusarium solani*. Its expression was significantly upregulated during the infection of black pepper (Piper nigrum) roots by *F. solani* cv. WN-1, indicating its potential role in pathogenicity. *FsGH28c* elicited cell death in *Nicotiana benthamiana* and modulated the expression of genes related to pathogenesis. *FsGH28c* exerts a positive influence on the pathogenicity of *F. solani*. The knockout of *FsGH28c* mutant strains markedly attenuated *F. solani* ’s virulence in black pepper plants. The knockout mutant strains decrease the ability of *F. solani* to utilize carbon sources. The *FsGH28c* deletion did not affect mycelial growth on PDA but did impact spore development. We identified a U-box protein, PnPUB35, interacting with FsGH28c using yeast two-hybrid and bimolecular fluorescence complementation assays. PnPUB35 conferred enhanced resistance to *F. solani* in black pepper through positive regulation. These findings suggest that FsGH28c may function as a virulence factor by modulating host immune responses through its interaction with PnPUB35.

## 1. Introduction

To breach the initial physical plant cell wall barrier, plant pathogens excrete cell wall-degrading enzymes (CWDEs), such as glycoside hydrolases [1,2]. While certain CWDEs augment pathogen virulence, others may trigger immune responses in plants [3,4]. For instance, the sucrose nonfermenting 1 gene (*VdSNF1*) of *Verticillium dahliae* [5], the pectate lyase gene *CcpelA* of *Colletotrichum coccodes* [6], and the pectate lyase gene *VdPEL1* of *V. dahliae* [7] were the CWDEs that play an important role in pathogen virulence. On the other hand, the secreted xyloglucanase BcXYG1 from *Botrytis cinerea* [8] and the xyloglucan-specific endoglucanase PsXEG1 from *Phytophthora sojae* [9] act as CWDEs to induce the immune responses of the host during pathogen infection. The symbiosis between pathogens and host plants is highly dynamic, with pathogenic pressure serving as a driving force for the evolution of plant immune mechanisms [10].

Glycoside hydrolases (GHs) are ubiquitous enzymes that cleave glycosidic bonds in cellulose, hemicellulose, and pectin components of plant cell walls [11]. In plants, GHs are key regulators of growth and development [12,13]. Pathogens exploit this mechanism by secreting GHs to facilitate the penetration of plant cell walls during infection [14,15]. For example, the soybean GH12 enzyme PsXEG1 from *P. sojae* promotes virulence, while the host protein GmGIP1 inhibits PsXEG1 and increases resistance [16]. Other examples include GH12 proteins VdEG1/VdEG3 and GH27 protein VdGAL4 from *V. dahliae*, which induce cell death and immune responses in *Nicotiana benthamiana* [14,17]. Polygalacturonases (PGs) in the GH28 family degrade pectin and regulate virulence and immunity in fungi like *B. cinerea*, *Lasiodiplodia theobromae*, and *V. dahliae* [15,18,19,20]. In *B. cinerea*, BcPG1 and BcPG2 play an important role in virulence [19,20]. In *Alternaria citri*, an endoPG mutant strain decreases the pathogen on citrus [21]. LtEPG1 of *L. theobromae* plays a crucial role in pathogenesis by facilitating pectin degradation and modulating the immune response, thereby promoting successful infection in *Vitis vinifera* [22]. The VdEPG1 mutant strain significantly decreases the pathogenicity of *V. dahliae* in cotton [15]. However, the molecular function of GH28 family members in *Fusarium solani* is still unclear.

The ubiquitin proteasome system contains the E1 ubiquitin-activating enzyme, E2 ubiquitin-conjugating enzyme, and E3 ubiquitin ligase enzyme [23]. E3 ligases are identified into four types: HECT, RING, U-box, and SCF in plants [24]. Plant U-box E3 ubiquitin ligases (PUBs) regulate growth, development, and stress responses in plants [25,26,27,28]. PUBs contained a conserved U-box domain [29], and 64 PUB members have been identified in Arabidopsis [30]. Some characterized PUBs act as negative regulators of immunity, like PUB22, PUB25/PUB26 in *Arabidopsis* and GhPUB17 in cotton, which suppress PAMP-triggered immunity or resistance to *V. dahliae* [31,32]. PUB25 and PUB26 also regulated *Arabidopsis* freezing tolerance and petal growth [33,34]. In cotton, GhPUB17 negatively regulated the cotton resistance to *V. dahliae*, and GhCyP3 can inhibit its E3 ligase activity [35]. However, other PUBs positively regulate defenses, including soybean GmSAUL1 (a bona fide U-Box E3 ligase) and apple MdPUB29, which confer resistance to pathogens by modulating cell death, immunity, and salicylic acid signaling [36,37]. Collectively, PUB E3 ligases serve as key regulators in the modulation of plant disease resistance.

The fungal pathogen *Fusarium solani* causes destructive wilt disease in the globally significant crop black pepper [38]. A previous study identified the *F. solani* strain WN-1 as the causal agent of black pepper *Fusarium* wilt in China [39]. Here, we found that the glycoside hydrolase gene FsGH28c is highly induced during WN-1 infection. Glycoside hydrolases like FsGH28c can function as virulence factors in other pathosystems. Additionally, plant U-box E3 ligases have emerged as key regulators of immunity during pathogen infection. However, the role of FsGH28c and its potential interplay with host U-box proteins in black pepper remains unknown. Investigating the function of FsGH28c and its interaction with host factors, such as U-box E3 ligases, is expected to yield critical insights into the molecular mechanisms underlying *Fusarium* wilt pathogenesis in black pepper. In this study, we characterize the virulence function of FsGH28c and identify a black pepper U-box protein that interacts with this pathogenicity factor. Our results shed light on how this glycoside hydrolase promotes virulence and engages host immunity regulators to facilitate *Fusarium* wilt disease.

## 2. Results

### 2.1. Identification and Expression Patterns of the GH28 Family Genes in F. solani

We identified nine glycoside hydrolase 28 (GH28) family members in the genome of *Fusarium solani* strain FSSC 5 MPI-SDFR-AT-0091 using HMMER searches with the Glyco_hydro_28 (PF00295) hidden Markov model from Pfam. The coding sequences of these GH28 genes ranged from 1083 to 1467 bp, encoding proteins of 360 to 488 amino acids with predicted isoelectric points from 5.34 to 9.03 and molecular weights from 37.163 to 54.062 kDa. All *F. solani* GH28 proteins contained a signal peptide (Table S3).

Phylogenetic analysis of GH28 family members from *F. solani* and other fungi, including *Verticillium dahliae* (11), *Fusarium oxysporum* (10), *Colletotrichum gloeosporioides* (19), *Botrytis cinerea* (20), *Phytophthora infestans* (25), *Phytophthora capsici* (17), and *Magnaporthe oryzae* (2), delineated three groups (Figure S1 and Table S4). Seven *F. solani* GH28s fell into Group A, while FsGH28c and FsGH28d were classified in Group B along with VdGH28-7/VdEPG1 from *V. dahliae* [15]. Group C contained no *F. solani* GH28 members. We also identified ten conserved motifs with distinct patterns among *F. solani* GH28 proteins (Figure S2) and variation in gene structure, specifically exon number (Figure S2). Figure S2b shows that FsGH28a, FsGH28b, FsGH28e, FsGH28f, FsGH28g, and FsGH28i contain motif 1 and are classified into the same clade. These results were consistent with those in phylogenetic analysis in Figure S1. In addition, Expression analysis by qRT-PCR revealed that *FsGH28c* (B0J15DRAFT_426735) was highly upregulated in both WN-1 spores and inoculated root samples compared to controls (Figure S3).

### 2.2. The FsGH28c Induced Cell Death in N. benthamiana

**Figure 1:**
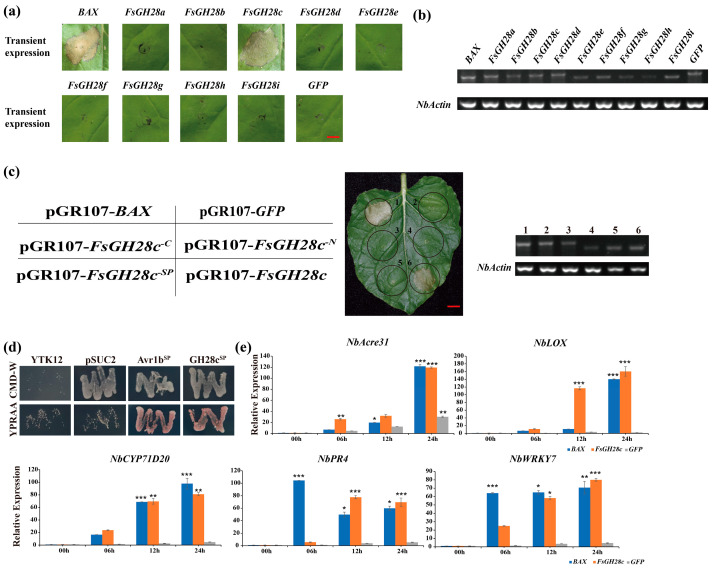
Identification of the cell death induced by *FsGH28c* in *Fusarium solani*: (**a**) Cell death assays for 9 *F. solani* genes in 6-week-old *N. benthamiana* leaves were performed by *Agrobacterium*-mediated transient expression. Leaves were imaged 6 days after infiltration with *Agrobacterium* carrying the *FsGH28* genes. BAX and GFP were used as the positive and negative controls, respectively. Scale bar = 0.5 cm. (**b**) Semi-quantitative reverse transcription PCR analysis of transiently expressed *FsGH28* genes in *N. benthamiana* leaves 48 h after infiltration. *NbActin* was used as the control. (**c**) Detection of the cell death activity of *FsGH28c*. The *FsGH28c* was transiently expressed in 6-week-old *N. benthamiana* leaves. Sq RT-PCR analysis showed that the *FsGH28c* was expressed in *N. benthamiana* leaves. The number 1 represents the sample that was only infiltrated with *BAX*, number 2 represents the sample that was only infiltrated with *GFP*, number 3 represents the sample that was infiltrated with truncation mutation *FsGH28c^-C^*, number 4 represents the sample that was only infiltrated with truncation mutation *FsGH28c^-N^*, number 5 represents the sample that was only infiltrated with *FsGH28c^-SP^*, and number 6 represents the sample that was only infiltrated with *FsGH28c*. *NbActin* was used as the control. Scale bar = 0.5 cm. (**d**) Confirmation of the function of the signal peptide of FsGH28c by yeast signal trap assay. Fusion of the functionality of the signal peptide of FsGH28c can grow on YPRAA medium. The functionality of the signal peptide of Avr1b was used as the positive control. (**e**) The qRT-PCR analysis of the pathogenesis-related genes. The blue, yellow, and grey columns represent the samples that were infiltrated by BAX, FsGH28c, and GFP at 00 h, 06 h, 12 h, and 24 h, respectively. *NbActin* was used as a control. Values represent means ± standard deviation of three replicates. * *p* < 0.05, ** *p* < 0.01, *** *p* < 0.001.

To determine if GH28 genes from *F. solani* can provoke an immune response in plants, a study utilizing *Agrobacterium*-mediated transient expression assays in *N. benthamiana* leaves was conducted, following the method described [15]. Transient expression analysis of nine GH28 genes revealed that specifically, *FsGH28c* could induce cell death in *N. benthamiana* leaves (Figure 1a). This was further corroborated by semiquantitative RT-PCR, which verified the transcription of all FsGH28 genes in the host plant (Figure 1b). Notably, *FsGH28c* lacking its signal peptide (*FsGH28c^-SP^*) failed to elicit cell death in the leaves (Figure 1c), and similarly, truncation mutants *FsGH28c^-C^* and *FsGH28c^-N^* were unable to trigger a hypersensitive response (HR) (Figure 1c). The signal peptide of FsGH28c, spanning amino acids 1 to 17, was listed in Table S2. A yeast signal trap system assay confirmed the functionality of the *FsGH28c* signal peptide (Figure 1d).

Furthermore, *N. benthamiana* leaves infiltrated with constructs expressing BAX, FsGH28c, and GFP were analyzed at 0, 6, 12, and 24 h post-infiltration (hpi) using RT-qPCR. The expression of pathogenesis-related marker genes *NbAcre3*1 [40], *NbCYP71D20* [41], *NbLOX*, *NbPR4* [42], and *NbWRKY7* [43] was significantly upregulated in response to *FsGH28c* treatment, as evidenced in Figure 1e. The expression levels of these genes were elevated in *FsGH28c*-infiltrated leaves compared to those infiltrated with GFP, indicating that *FsGH28c* activates immune responses in plants.

### 2.3. FsGH28c Is Required for the Utilization of Certain Carbon Sources

**Figure 2:**
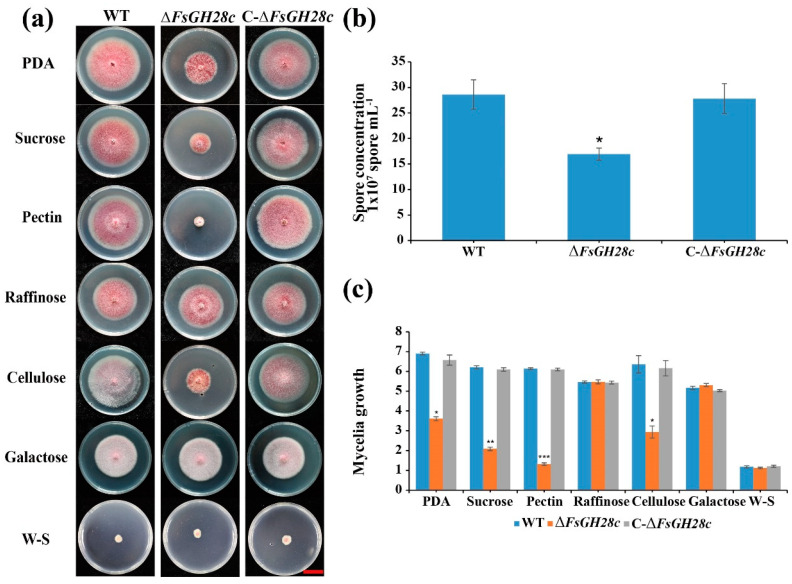
FsGH28c plays an important role in utilizing carbon source: (**a**) Phenotype analysis of the wild-type, ∆*FsGH28c*, and C-∆*FsGH28c* strains grown on PDA and Cazpek Dox medium with sucrose, galactose, pectin, raffinose, or cellulose as the sole carbon source for 7 days. Scale bar = 3 cm. (**b**) The spore concentration of the wild-type, ∆*FsGH28c*, and C-∆*FsGH28c* strains grown in liquid Czapek Dox medium for 3 days. Values represent means ± standard deviation of three replicates. The asterisks represent statistical differences performed by a *t*-test (* *p* < 0.05) in comparison with the wild-type strains. (**c**) The mycelial growth of the wild-type, ∆*FsGH28c*, and C-∆*FsGH28c* strains on Cazpek Dox medium at 7 days. Values represent means ± standard deviation of three replicates. The measurement unit represents centimeters. The asterisks represent statistical differences performed by a *t*-test (* *p* < 0.05, ** *p* < 0.01, *** *p* < 0.001) in comparison with the wild-type strains.

To investigate the role of *FsGH28c* in virulence, we generated knockout mutant strains *∆FsGH28c* and complemented strains C-∆*FsGH28c* using the homologous recombination method as previous study (Figure S4a,b) [15]. Compared to the wild-type WN-1 (WT), the ∆*FsGH28c* strains showed reduced mycelial growth and conidia counts, and C-∆*FsGH28c* strains showed increased ability of mycelial growth and conidia production (Figure 2).

To investigate the role of *FsGH28c* in using different carbon sources, the mycelial radial growth of WT, ∆*FsGH28c*, and C-∆*FsGH28c* strains was assessed on Czapek Dox solid medium supplemented with various carbon sources, including sucrose, galactose, raffinose, pectin, or cellulose. The Δ*FsGH28c* mutants displayed significantly impaired growth in a medium containing sucrose, pectin, or cellulose compared to WT, especially in a pectin medium (W-S: without sucrose; Figure 2a,c). No difference was observed between strains in raffinose or galactose medium. These results suggest that *FsGH28c* plays a crucial role in the vegetative growth of *F. solani* and the utilization of specific carbon sources, particularly pectin.

### 2.4. FsGH28c Is Required for Microconidia Development

**Figure 3:**
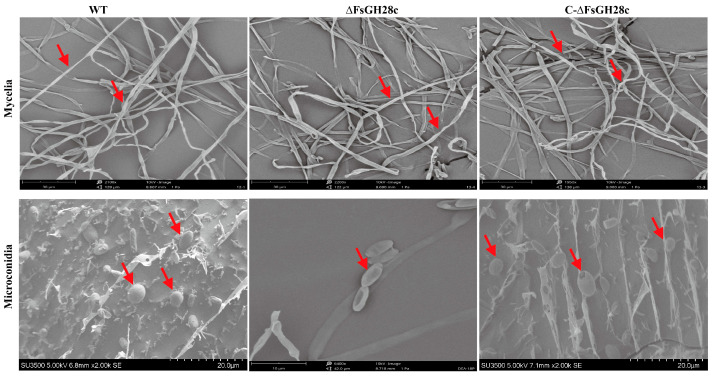
Observation of the mycelium and conidia on a scanning electron microscope: Mycelium of the wild-type, ∆*FsGH28c*, and C-∆*FsGH28c* strains grown on PDA medium for 5 days, and microconidia of the wild-type, ∆*FsGH28c*, and C-∆*FsGH28c* strains incubated in Cazpek Dox for 3 days were observed via scanning electron microscope, respectively. The red arrows point to the mycelium and microconidia of each strain. Scale bar = 20 μm.

Scanning electron microscopy revealed no apparent differences in mycelial morphology between WT, Δ*FsGH28c*, and C-Δ*FsGH28c* strains grown on PDA medium (Figure 3). However, microconidia of Δ*FsGH28c* mutants were irregularly shaped and hollow compared to the relatively plump, smooth microconidia observed in WT and C-Δ*FsGH28c* strains. These results indicate that *FsGH28c* plays an important role in *F. solani* conidial development.

### 2.5. The FsGH28c Is Required for Full Virulence on Black Pepper

**Figure 4:**
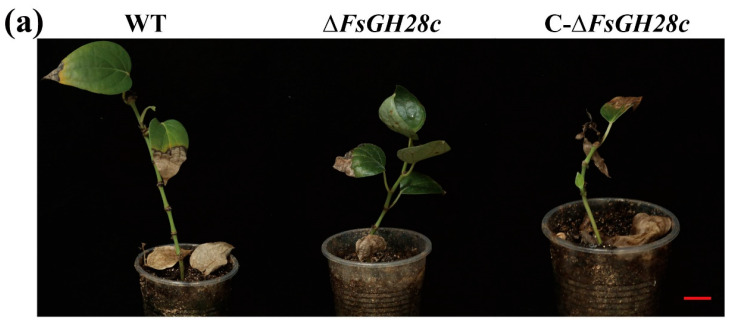

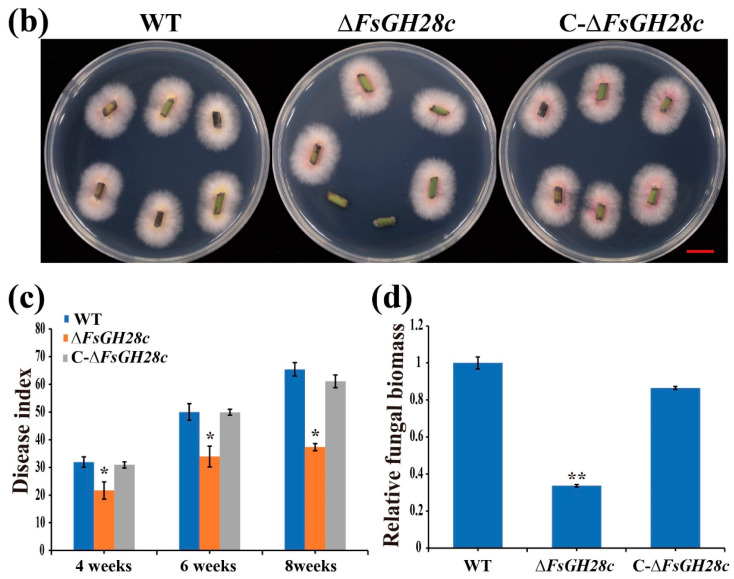
FsGH28c plays a positive role in the virulence of *Fusarium solani*: (**a**) Disease symptoms of black pepper after the wild-type, ∆*FsGH28c*, and C-∆*FsGH28c* strains infection. Photographs were taken at 8 weeks after fungal inoculation. Each treatment has more than 30 black peppers. Each treatment has three replicates. Scale bar = 3 cm. (**b**) Re-isolation of *F. solani* WN-1 strains from the stem of inoculated black pepper plants at 26 °C for 3 days. Scale bar = 1.5 cm. (**c**) Disease index of black pepper plants at 4 weeks, 6 weeks, and 8 weeks after the wild-type, ∆*FsGH28c*, and C-∆*FsGH28c* strains infection. (**d**) Relative fungal biomass in stems of black pepper after the wild-type, ∆*FsGH28c*, and C-∆*FsGH28c* strains infection at 8 weeks. Values represent means ± standard deviation of three replicates. The asterisks represent statistical differences performed by a *t*-test (* *p* < 0.05, ** *p* < 0.01) in comparison with the wild-type strains.

To examine ∆*FsGH28c* virulence, *Piper nigrum* cv. *Reyin*-*1* plants were inoculated with WT, Δ*FsGH28c*, and *C-*Δ*FsGH28c* strains. Black pepper plants inoculated with Δ*FsGH28c* mutants displayed only mild wilting symptoms compared to WT and complemented strains (Figure 4a). Fungal recovery assays revealed that Δ*FsGH28c* mutants still colonized black pepper tissues but formed fewer colonies than WT and C-Δ*FsGH28c* (Figure 4b). Disease index analysis showed that pepper infected with Δ*FsGH28c* had significantly reduced disease compared to WT and complemented infection (Figure 4c). qPCR quantification confirmed that Δ*FsGH28c* mutants accumulated lower fungal biomass in inoculated pepper stems versus WT and complemented strains (Figure 4d). Together, these results demonstrate that *FsGH28c* is important for *F. solani* virulence in black pepper.

### 2.6. FsGH28c Interacts with PnPUB35

**Figure 5:**
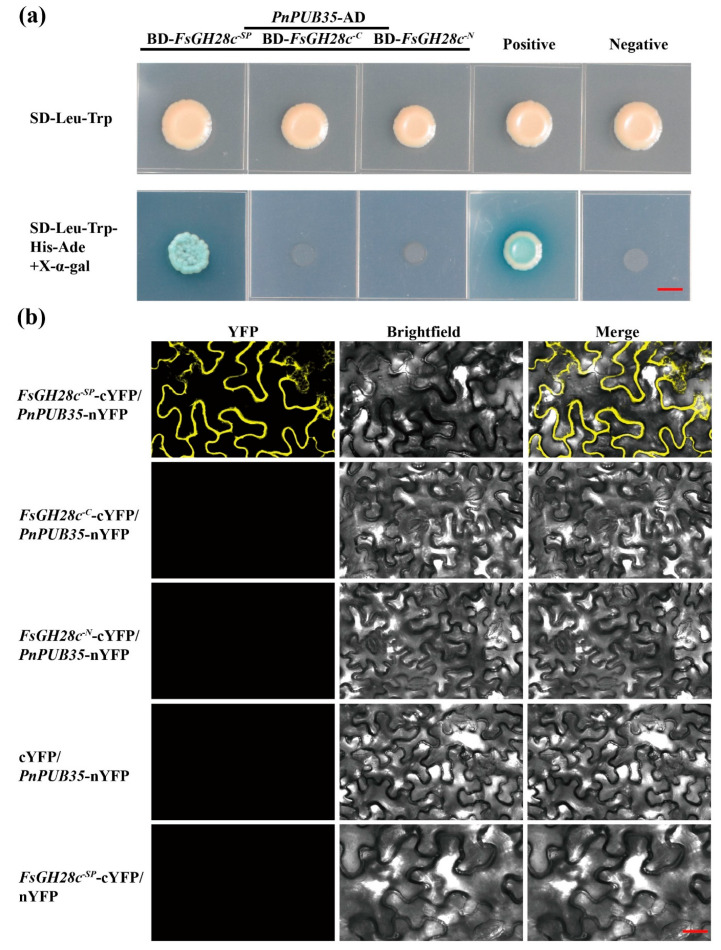
*FsGH28c* interacts with the *PnPUB35*: (**a**) The interaction between *FsGH28c* and *PnPUB35* was confirmed by Yeast two-hybrid assays. *FsGH28c^-SP^* was an SP truncation mutation, *FsGH28c^-C^* was an N-terminal truncation mutation, and *FsGH28c^-N^* was a C-terminal truncation mutation. Scale bar = 0.3 cm. (**b**) BiFC assay showing that the interaction between *PnPUB35*-nYFP and *FsGH28c^-SP^*-cYFP formed a functional YFP in the plasma membrane. Scale bar = 100 µm.

To further study the regulatory network of *FsGH28c*, the pGBKT7-*FsGH28c^SP^* was constructed to be used as a bait vector via Y2H assay to screen the *F. solani*-inoculated black pepper root cDNA library. Fourteen blue colonies on the QDO/X/A plate were selected to perform yeast colony PCR (Figure S5a). The results showed that a positive interaction was caught (Figure S5b). Through sequencing and blasting, Pn6.961 was identified (Table S5). Pn6.961 was identified to interact with *FsGH28c* in the Y2HGold yeast strain (Figure 5a). Pn6.961 contained a U-box domain with 49% homology with AtPUB35 (AT4G25160) (Figure S6a,b). So, Pn6.961 was named as PnPUB35 in this study. *PnPUB35* was highly expressed in the stem of black pepper (Figure S6c). The *PnPUB35* expression was upregulated in the root of black pepper under the WN-1 infection (Figure S6d). Bimolecular fluorescence complementation (BiFC) assays were performed to test the interaction in vivo. The *Agrobacterium* of *FsGH28c^-SP^*-cYFP and *PnPUB35*-nYFP were co-infiltrated into *N. benthamiana* leaves. Under laser confocal scanning microscopy, yellow fluorescent protein (YFP) localization was observed at the plasma membrane (Figure 5b). The control treatments, co-infiltrated with *FsGH28c^-C^*-cYFP and *PnPUB35*-nYFP, *FsGH28c^-N^*-cYFP and *PnPUB35*-nYFP, cYFP with PnPUB35-nYFP, and FsGH28c^-SP^-cYFP with nYFP, did not show fluorescence (Figure 5b). The results showed that the interaction between FsGH28c and PnPUB35 emerged in the *N. benthamiana* plasma membrane. FsGH28c-YFP was located in the plasma membrane, and PnPUB35-YFP was located in the nucleus in *N. benthamiana* leaves (Figure S7). These results showed that FsGH28c interacts with PnPUB35 in vitro and in vivo.

### 2.7. PnPUB35 Plays a Positive Role in Resistance to F. solani

**Figure 6:**
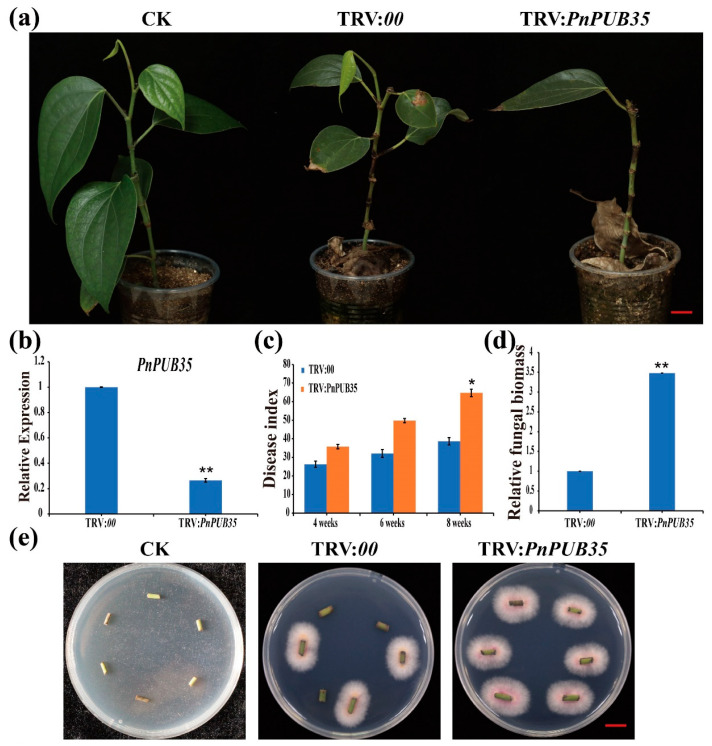

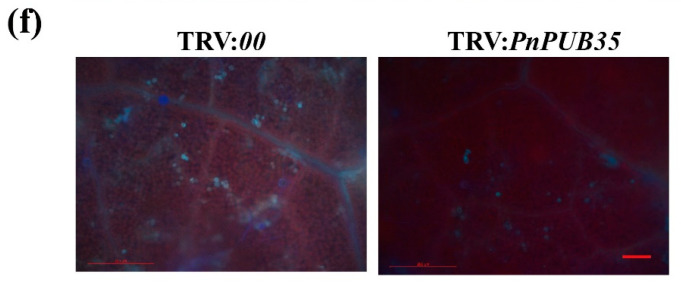
PnPUB35 positively regulates black pepper resistance against *Fusarium solani*: (**a**) Disease symptoms of the black pepper plants after WN-1 infection. Photographs were taken at 8 weeks after inoculation. Scale bar = 2 cm. (**b**) The expression level of *PnPUB35* in the treatment plants. Total RNA was isolated from roots at 21 days post-agroinfiltration. *PnMLF1* was used as the reference. Each experiment was performed using three independent replicates (** *p* < 0.01). (**c**) Disease index of the treatment plants at 4 weeks, 6 weeks, and 8 weeks after inoculation with WN-1. Each experiment was performed using three replicates (* *p* < 0.05). (**d**) qPCR analysis of the relative fungal biomass in stems of the treatment plants at 8 weeks after WN-1 inoculation. Each experiment was performed using three replicates. Differences between groups were compared using the *t*-test (** *p* < 0.01). (**e**) Re-isolation of *F. solani* from the stem of inoculated black pepper plants at 26 °C for 3 days. Scale bar = 1 cm. (**f**) Callose deposition in leaves of the treatment plants at 6 weeks after WN-1 inoculation. Leaves were imaged on fluorescence microscopy. Scale bar = 200 μm.

To further investigate the function of PnPUB35 in resistance to *F. solani*, black pepper plants were infiltrated with the VIGS constructs TRV: *PnPUB35* and TRV:*00*. The silenced seedlings were infected with *F. solani* WN-1. The TRV: *PnPUB35* plants showed more severe symptoms than the TRV:*00* plants (Figure 6a). RT-PCR and qRT-PCR tested the gene-silencing efficiency of treatment plants. The results showed that *PnPUB35* was successfully silenced (Figure 6b and Figure S8). The disease index (DI) of black pepper was investigated at 4, 6, and 8 weeks after inoculation. The results showed that the DI of *PnPUB35-*silenced plants infected by WN-1 strains was about 64.71. While the DI of TRV:*00* plants was approximately 38.63 at eight weeks (Figure 6c). Fungal biomass assays in treatment plant roots were performed by qPCR. The results showed that the fungal biomass in *PnPUB35-*silenced plants was more than that in TRV:*00* plants (Figure 6d). Fungal recovery and callose deposition assays showed that *PnPUB35-*silenced plants showed more susceptible to *F. solani* WN-1 than TRV:*00* plants (Figure 6e,f).

**Figure 7:**
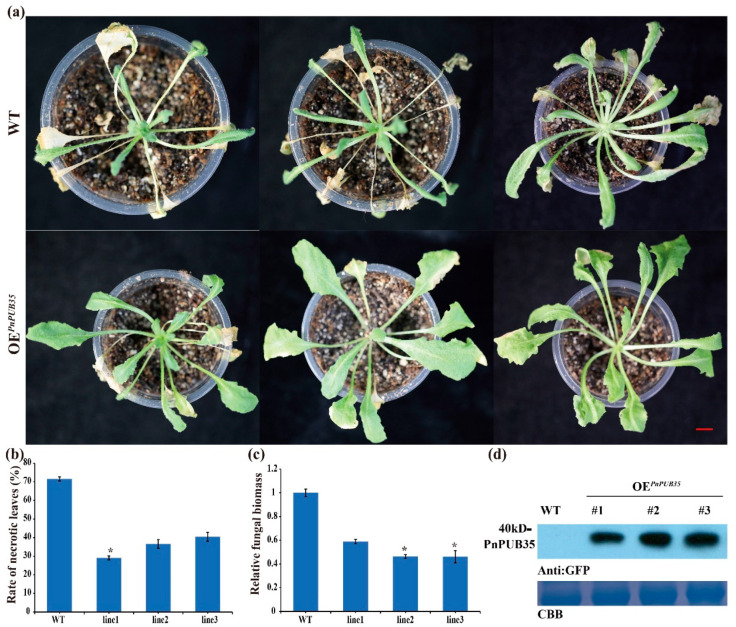
Overexpression of *PnPUB35* enhances *Arabidopsis thaliana* resistance to *Fusarium solani*: (**a**) Disease symptoms of the *A. thaliana* plants after WN-1 infection. WT represents wild-type *A. thaliana* plants, and OE*^PnPUB35^* represents *PnPUB35* overexpression transgenic *A. thaliana* line plants. Photograph was taken 14 days after inoculation with WN-1. Scale bar = 1 cm. (**b**) The necrotic leaves rate of *A. thaliana* plants at 14 days after WN-1 infection. Differences between groups were compared using the *t*-test (* *p* < 0.05). (**c**) qPCR analysis of the relative fungal biomass in leaves of the treatment plants at 14 days after WN-1 inoculation. Each experiment was performed using three replicates. Differences between groups were compared using the *t*-test (* *p* < 0.05). (**d**) Western blot analysis of PnPUB35 expression in *A. thaliana* plants. CBB was used as the control.

Then, the *PnPUB35-*overexpression *Arabidopsis* plants were inoculated with *F. solani* WN-1. The *PnPUB35-*overexpression *Arabidopsis* plants showed more resistance to *F. solani* WN-1 (Figure 7a,b). In addition, less fungal biomass was detected in overexpression plants (Figure 7c). PnPUB35 was successfully detected in overexpression plants by Western blotting (Figure 7d). These results suggested that PnPUB35 contributes positively to enhancing black pepper’s resistance to *F. solani*.

## 3. Materials and Methods

### 3.1. Fungus and Plant

The *F. solani* strain WN-1 was propagated following the methodology outlined by our previous study [44,45]. This fungal strain was incubated in solid potato dextrose agar (PDA) for seven days at 25 °C, and the microconidia were inoculated into yeast extract peptone dextrose (YPD, 2% dextrose, 2% peptone, 1% yeast extract) broth and germinated at 28 °C at 160 rpm. Cultivation of black pepper plants (*Piper nigrum* cv. *Reyin*-*1*) occurred in a greenhouse, maintained at 28 °C, under a controlled photoperiod of 16 h light and 8 h dark, following the protocol described by [39] Liu et al. Similarly, *A. thaliana* seedlings were cultivated in a growth chamber maintained at 24 °C under the same photoperiod of 16 h light and 8 h dark. In contrast, tobacco plants (*Nicotiana benthamiana*) were cultivated for six weeks at 25 °C in a greenhouse under a 14 h light and 10 h dark photoperiod to facilitate transient expression studies.

### 3.2. PVX Vector Construction and Transient Expression Assay

The full-length coding sequences of the *FsGH28* were obtained through amplification from *F. solani* cDNA by specific primers. These sequences were then cloned into the PVX vector pGR107 at the *Cla*I-*Xma*I restriction sites utilizing the Hieff Clone^®^ Universal II One step Cloning Kit (Yeasen, Shanghai, China). Subsequently, the recombinant PVX plasmids were introduced into *Agrobacterium tumefaciens* strain GV3101 for transient expression studies in *N. benthamiana* leaves. Expression levels were assessed using BAX as a positive control and green fluorescent protein (GFP) as a negative control [46]. Post-*Agrobacterium* infiltration, leaf samples were collected at specified time points (0, 6, 12, and 24 h) for RNA extraction. Symptoms were documented at six days post-infiltration (dpi). Each experimental replicate comprised three leaves from three distinct plants, with the entire procedure repeated thrice. The primer details are listed in Table S1.

### 3.3. Yeast Signal Sequence Trap System

The verification of the *FsGH28c* signal peptide’s functionality was assessed following the previously established protocol [47]. The signal peptide in question, along with the Avr1b signal peptide as a positive control, was respectively cloned into the pSUC2 vector. These constructs, designated as pSUC2-*FsGH28c*^SP^ and pSUC2-Avr1b^SP^, underwent transformation into the yeast strain YTK12 and were subsequently selected on CMD-W medium. Transformants exhibiting positive growth were cultured in YPRAA (YPR Agar Medium, 1% yeast extract, 2% peptone, 2% raffinose, 2% agar) medium containing antimycin A. For negative control comparisons, the yeast strain was transformed with the empty pSUC2 vector. The specific primers utilized in these experiments are detailed in Table S1.

### 3.4. The Fungal Transformation Constructs

Homologous recombination facilitated the creation of the ∆*FsGH28c* mutants and their respective complementary strains, following the methodology reported [15]. Approximately 1.1 kb of upstream and downstream flanking sequences of *FsGH28c*, along with the hygromycin resistance cassette (HPH), were amplified from the genomic DNA of WN-1. These amplified sequences were subsequently integrated into the knockout vector B303 using the In-Fusion HD Cloning Kit (Clontech, TaKaRa, Mountain View, CA, USA) due to their overlapping regions [15]. For the complementary strains, the construction of plasmids was executed as per the protocol delineated [48], involving the insertion of a 1.5 kb promoter of *FsGH28c* and its complementary DNA into the pCAMBIA1302-Neo vector, which contains the geneticin resistance cassette. Primer details are provided in Table S1.

### 3.5. Fungal Transformations

The resultant constructs, B303-HPH-*FsGH28c* for knockout and pCAMBIA1302-Neo- *FsGH28c* for complementation, were employed to transform *F. solani* protoplasts, adhering to the procedures established [45,49]. Approximately 1 × 10^8^ microconidia were inoculated into YPD broth and germinated at 28 °C at 160 rpm overnight. The germlings were mixed with the enzyme mixture of Lysing Enzymes (5 mg/mL, from *Trichoderma harzianum*), Driselase (5 mg/mL), and Chitinase (100 µg/mL, from *Streptomyces griseus*). The mixture was incubated at 28 °C for 2 h at 50 rpm. The formation of protoplasts was monitored by the microscope. Protoplasts were collected and washed twice in STC50 buffer (1.2 M Sorbitol, 10 mM CaCl_2_, 10 mM Tris HCl pH 7.5). Each 200 µL of 2 × 10^7^ protoplast suspension was used for each transformation.

More than 10 µg of the construct template was combined with protoplasts. This solution was placed on ice for 20 min. Then, 1.5 mL of the 60% PEG solution (60% (*w*/*v*) PEG MW 3350, 50 mM CaCl_2_, 50 mM Tris HCl pH 7.5) was added and mixed by gently swirling and incubated at room temperature for 20 min; 2 mL of STC50 was added to each transformation and incubated at room temperature for 2 min; and 8 mL of STC50 was added to each transformation and incubated at 28 °C for 18 h without light. The final mixture was plated onto PDA and incubated at 28 °C. The selection of transformants relied on vector-specific antibiotic resistance and was subsequently confirmed using RT-PCR. Primer details are provided in Table S1.

### 3.6. Carbon Source Utilization Assays

To analyze carbon source utilization of WT, *∆FsGH28c*, and C-∆*FsGH28c* strains, sucrose (30 g/L), pectin (10 g/L), raffinose (10 g/L), cellulose (5 g/L), or galactose (10 g/L) were mixed in Czapek Dox medium without sucrose for 7 days, as described previously. The colony growth diameter was measured by the cross method. The experiment was repeated six times.

### 3.7. Pathogenicity

This study utilized various strains, including deletion, complementary, and wild-type strain WN-1, cultured following the protocols we previously described [39]. The conidial suspensions were adjusted to a standardized concentration of 1 × 10^7^ spores mL^−1^. To evaluate the virulence of these strains, *Piper nigrum* cv. *Reyin*-*1* plants were employed. These plants were cultivated in Hoagland’s solution under controlled conditions, with a 16 h light/8 h dark photoperiod at 28 °C. Upon development of four true leaves, the plant seedlings underwent a 30 min immersion in the spore suspension, subsequently being replanted in sterilized soil to continue growth and facilitate observation. Root and leaf samples were collected at 0, 6, 12, and 24 h post-inoculation for analysis. The WT WN-1 spores that inoculated the black pepper after 0, 6, 12, and 24 h were collected and centrifuged for qRT-PCR analysis of *FsGH28* genes. Fungal recovery from black pepper stem sections was conducted 45 days post-inoculation, adhering to the methodology of [39] Liu et al. Fungal biomass quantification followed the procedure detailed [38]. The assessment of plant wilt was quantified using a severity scale ranging from 0 (no symptoms) to 4 (1 = ≤33%, 2 = >33% and ≤66%, 3 = > 66% and ≤99%, and 4 = 100%), with intermediate values indicating increasing severity of symptoms.

The disease index (DI) for each plot was determined using the following formula:

*DI* = [(0 × n_0_ + 1 × n_1_ + 2 × n_2_ + 3 × n_3_ + 4 × n_4_)/4 × n] × 100% (1)

where n_0_–n_4_ represent the number of plants manifesting each disease rating scale, and *n* is the total number of plants evaluated per plot. Sixty days post-inoculation, total DNA was extracted from the seedling stems for quantitative PCR analysis, with *PnMLF1* (Myeloid leukemia factor 1, Pn17.1212) serving as the reference gene for black pepper, following the methodology [38].

### 3.8. Scanning Electron Microscope Assay

Scanning electron microscope (SEM) observations of morphological characteristics of the mycelia and microconidia of WT, ∆*FsGH28c*, and C-∆*FsGH28c* strains were performed by using the method as previously described [39]. The mycelia of WT, ∆*FsGH28c*, and C-∆*FsGH28c* strains were washed with double-distilled water from 5-day-old PDA and centrifuged at 2500× *g* for 10 min, respectively. And the microconidia of WT, ∆*FsGH28c*, and C-∆*FsGH28c* strains from YPD broth (germinated at 28 °C at 160 rpm for 3 days) were centrifuged at 2500× *g* for 10 min, respectively. The samples were treated with 0.5% DMSO. The mycelia and microconidia were first immersed in 2.5% glutaraldehyde and stored at 4 °C overnight. Subsequently, the samples were washed in 1× PBS buffer (Solarbio, pH 7.2) and dehydrated using a graded ethanol series of 30%, 50%, 75%, 95%, and 100%. Following critical point drying, the dried samples were observed using scanning electron microscopy (Hitachi SU-3000, Tokyo, Japan), respectively.

### 3.9. Yeast Two-Hybrid Assays

The construction of a cDNA library from black pepper roots post-inoculation with *F. solani* WN-1 was accomplished utilizing the Matchmaker Gold Yeast Two-Hybrid (Y2H) System (Clontech, Cat. No. 630489, TaKaRa, Mountain View, CA, USA) for the purpose of Y2H screening. Within this framework, the *FsGH28c^-SP^* gene was integrated into the pGBKT7 vector as bait to facilitate the screening of interacting proteins from the cDNA library. To substantiate the interaction between *FsGH28-3* and the newly identified protein PnPUB35, the pGBKT7 plasmid harboring the *FsGH28c^-SP^* fusion was introduced into the Y2H yeast strain. Concurrently, the pGADT7 plasmid containing PnPUB35 was transformed into the Y187 yeast strain using the specified Transformation System (Clontech, Cat. No. 630489, TaKaRa, Mountain View, CA, USA). Following transformation, mating of the strains was executed, and selection was carried out on SD-Leu-Trp and SD-Leu-Trp-His-Ade media supplemented with X-α-gal, adhering to the protocols delineated in the Clontech manual (Cat. No. 630489, TaKaRa, Mountain View, CA, USA). The primers utilized are enumerated in Table S1.

### 3.10. BiFC Analysis and Subcellular Localization

In the study, to elucidate the in vivo interaction between FsGH28c and PnPUB35, bimolecular fluorescence complementation (BiFC) analysis was used, as previously described [50]. The coding sequences for *FsGH28c^-SP^* (18–1083 bp), *FsGH28c^-C^* (18–540 bp), and *FsGH28c^-N^* (541–1083 bp) were cloned into the vectors pXY104cYFP, and the coding sequences of *PnPUB35* were cloned into the vectors pXY106nYFP, respectively. Subsequently, *A. tumefaciens* strain GV3101 harboring the recombinant constructs *FsGH28c^-SP^*-cYFP and *PnPUB35*-nYFP was co-infiltrated into *N. benthamiana* leaves to facilitate in planta expression. To ascertain the subcellular localization of the proteins, fusion constructs of FsGH28c and PnPUB35 with the reporter gene YFP were generated in the vector pCAMBIA2300-YFP. The resulting positive bacterial cultures were then infiltrated into *N. benthamiana* leaves. Fluorescence signals of YFP were detected at 488 nm, using a confocal microscope 48 h post-infiltration. The details of the primers utilized are delineated in Table S1.

### 3.11. Virus-Induced Gene Silencing (VIGS)

To further investigate the functions of PnPUB35, the VIGS method was utilized. The coding sequence (CDS) of *PnPUB35*, amplified from *Piper nigrum* cv. *Reyin*-*1*, was cloned into the tobacco rattle virus (TRV) vector pYL156, yielding the construct TRV: *PnPUB35*, following the methodology [15]. The TRV:00 construct served as the negative control. These TRV constructs were infiltrated into the leaves of black pepper seedlings, adhering to the protocol established [51]. To evaluate the silencing efficiency of *PnPUB35*, new leaves from both TRV: *PnPUB35* and *TRV:00* lines were examined. Plants in which *PnPUB35* was successfully silenced underwent inoculation with the wild-type *F. solani* strain WN-1, consistent with procedures previously detailed [39]. Disease Index (DI), fungal recovery, and fungal biomass assays were conducted in accordance with the methods described by [15] Liu et al.

### 3.12. Callose Deposition

Callose deposition was evaluated using aniline blue staining, following the method described [52]. Leaves were first destained in a 3:1 ethanol/acetic acid solution for 3 h, then sequentially treated with 70% and 50% ethanol for 2 h each. Subsequently, the leaves were rinsed in distilled water for 12 h and further destained in 10% (w/v) NaOH for 2 h. Finally, the samples were stained with 0.01% (w/v) aniline blue and visualized under fluorescence microscopy. Primer sequences are provided in Table S1.

### 3.13. Arabidopsis Transformation

The CDS of *PnPUB35* was subcloned into the plant binary vector pCAMBIA2300, driven by the CaMV 35S promoter. This recombinant construct was then introduced into *A. tumefaciens* strain GV3101. Transformants exhibiting positive integration were selected for the transformation of *Arabidopsis* using the floral dip method [53]. Screening of the progeny was conducted on Murashige and Skoog (MS) medium containing 50 mg L^−1^ of the selective agent. The T3 transgenic *Arabidopsis* lines were validated through PCR analysis and subsequently utilized in pathogenicity assays. The specific primers used in these experiments are listed in Table S1.

### 3.14. Protein Extraction and Western Blotting

Protein extraction from *N. benthamiana* and *A. thaliana* leaves post-treatment followed the protocol outlined [46]. Treated leaves were pulverized in liquid nitrogen and homogenized with an equal volume of protein isolation buffer (62.5 mmol·L^−1^ Tris–HCl, pH 7.4, 10% glycerol, 2% SDS, 1 mmol·L^−1^ PMSF, 2 mmol·L^−1^ EDTA, 100 mmol·L^−1^ DTT). The homogenate was centrifuged at 13,000× *g* for 10 min at 4 °C, and added with one-fourth volume of loading buffer (50 mmol·L^−1^ Tris, pH 6.8, 200 mmol·L^−1^ DTT, 2% SDS, 0.1% bromophenol blue, 10% glycerol). The resulting supernatant was mixed with protein sample buffer and heated at boiling temperature for 5 min and was stored at −20 °C.

Total proteins were then separated via 10% sodium dodecyl sulfate-polyacrylamide gel electrophoresis (SDS-PAGE) (160 V, 80 min), and transferred onto polyvinylidene difluoride (PVDF) membranes (100 V, 60 min) for further analysis. After the membrane was incubated with antibodies, the intensity of signal was produced by the super-sensitivity ECL luminescent solution and detected by a chemiluminescence imager (Beijing Saizhi, MiniChemi610) [54]. The band detected by anti-GFP antibody was used as loading control.

### 3.15. Nucleic Acid Extraction and Expression Analysis

Total RNA was extracted using the RNAprep Pure Plant Plus Kit (Polysaccharides & Polyphenolics-rich) from TransGen Biotech (Beijing, China), following the manufacturer’s protocol. Similarly, total DNA was isolated with the Fungal DNA Kit from Omega Bio-tek (Norcross, GA, USA), according to the provided instructions. First-strand cDNA synthesis was performed using the All-in-One First-Strand cDNA Synthesis Super Mix for qPCR (TransGen Biotech, Beijing, China). Both RT-PCR and qPCR were carried out following the methodologies described [52].

### 3.16. Bioinformatics Analysis

The Hidden Markov Model (HMM) for Glyco_hydro_28 (PF00295) was retrieved from the Pfam database to facilitate the identification of GH28 family members, applying an E-value threshold of less than 1, using HMMER version 3.1b2 software against fungal proteomes [55,56]. Subsequent to multiple sequence alignments executed with MUSCLE, as described, members of the GH28 family were used to construct a maximum likelihood (ML) phylogenetic tree employing MEGA X software (version 10.2.2) [57,58]. The fungal genome databases, including *Fusarium solani*, *Verticillium dahliae*, *Magnaporthe oryzae*, *Phytophthora capsici*, *Phytophthora infestans*, *Fusarium oxysporum*, *Botrytis cinerea*, and *Colletotrichum gloeosporioides*, were accessed and downloaded from the NCBI website (http://www.ncbi.nlm.nih.gov. URL (accessed on 22 April 2024)). The genome annotation information used in this study is in Table S2.

## 4. Discussion

The pathogen *F. solani* is phylogenetically classified within the *F. solani* species complex (FSSC) [59]. It is a soilborne fungal pathogen capable of colonizing both living and dead plant tissues, with the ability to persist in the soil for extended periods [60,61]. Previous studies showed that *F. solani* can infect many crops [39,62,63,64,65]. Certain strains of *F. solani* have been reported to pose risks to human and animal health [66,67,68]. Compared to humans and animals, plants have no specialized immune system. When phytopathogens infect the plants, plants induce sophisticated defense systems, PTI and ETI, to protect themselves from pathogens [69]. Nevertheless, the plant–pathogen interaction system, especially between *F. solani* and black pepper, has not yet been elucidated. In the present study, we detected that *FsGH28c* could induce cell death and activate plant immune responses, functioning as a key virulence factor that plays a pivotal role in fungal pathogenicity during infection, and interacted with PnPUB35 of the black pepper host.

Previous studies showed that GH members of phytopathogens were identified in many phytopathogens. Among oomycete plant pathogens, many GH12 proteins (6 to 12 per species) within the *Phytophthora* genus were identified [9]. In *V. dahliae* V991, six GH12 proteins were identified and further studied [70]. Four genes belonging to the GH28 family were found in *Lasiodiplodia theobromae* [22]. Bioinformatics analysis identified five proteins in *V. dahliae* (designated as VdGAL1 through VdGAL5) containing glycoside hydrolase family 27 (GH27) domains [14]. In our research, nine glycoside hydrolase-28 family members were found in the *F. solani* genome. Like the XEG1 [9], VdEG1 and VdEG3 [70], LtEPG1 [22], and VdGAL4 [14], the expression level of *FsGH28c* was significantly elevated in the spore suspension of *F. solani* WN-1 and in infected black pepper roots. This finding suggests that FsGH28c is a key contributor to the virulence of *F. solani* WN-1.

The agroinfiltration method is a widely employed technique for investigating plant resistance mechanisms, pathogen virulence, and the functional roles of effector genes in various plant species [71,72], especially to identify the fungal virulence factors that can induce the hypersensitive response [15,73,74]. Many GH family members can trigger hypersensitive responses, like XEG1, VdEG1, VdEG3, LtEPG1, FoEG1 [75], and VdGAL4. Previous studies showed that *F. solani* infects black pepper primarily through the roots and subsequently spreads systemically to the leaves [39]. These studies suggest that agroinfiltration of plant leaves can be used in *F. solani*. In our study, the *Agroinfiltration* assay of nine GH28 members of *F. solani* in *N. benthamiana* leaves showed that the FsGH28c can induce cell death. Furthermore, the pathogenesis-related genes *NbAcre31*, *NbCYP71D20*, *NbLOX*, *NbPR4*, and *NbWRKY7* were activated by infiltration of FsGH28c in *N. benthamiana* leaves. Pathogens secreted virulence compounds to invade the host and cause plant disease symptoms [76]. However, in the yeast signal sequence trap system, several proteins secreted by pathogens have been identified, including VdCUT11 [17], LtEPG1 [22], FoEG1 [75], VdHP1 [77], and FoRnt2 [78]. In the current study, the signal peptide of FsGH28c was functional via the yeast signal sequence trap system assay. In addition, *FsGH28c* was highly expressed in infected black pepper roots. These suggested that *FsGH28c* was most likely secreted into the extracellular space during the infection of the host plant. Moreover, lacking the signal peptide, FsGH28c^-SP^ did not trigger cell death in *N. benthamiana* leaves. These results suggest that FsGH28c may be secreted by *F. solani* and trigger plant defense responses during infection.

To attack the plant cell wall, pathogenic fungi can degrade the host cell walls. *FoERG3* is associated with the biosynthesis of ergosterol, a crucial component of the fungal cell membrane that plays a key role in maintaining membrane structure and function. The deletion of *FoERG3* disrupted ergosterol biosynthesis, inhibited mycelial growth, and markedly reduced the utilization efficiency of various carbon sources [79]. Rho2 plays a critical role in the development, stress response, and pathogenicity of *F. oxysporum*. The growth of Δ*rho2* was destroyed under cell wall disturbing stress and high-temperature stress [44]. GHs can hydrolyze cleaving glycosidic bonds in oligo- or polysaccharides, substrates that contain cellulose, hemicellulose, and pectin [11]. Δ*VdGAL4* strains exhibited significantly reduced colony growth on media supplemented with raffinose and sucrose, alongside a marked decrease in the ability of VdGAL4 to hydrolyze α-1,6 glycosidic bonds [14]. VdEPG1 can hydrolyze pectin to produce galacturonic acid (Gal-A). When the *VdEPG1* gene was silenced, the mutant strains demonstrated significantly reduced growth in media containing pectin [15]. In our research, mycelial growth of Δ*FsGH28c* strains was reduced considerably in a medium containing pectin, which indicates that *FsGH28c* participated in the utilization of pectin in the host cell wall during infection. Numerous studies have demonstrated that conidia and mycelium serve a critical role in the pathogenicity of pathogens in plants. VdNoxB, VdPls1, and VdSte11 deleted strains produce defective hyphopodia and display significantly decreased virulence [80,81]. The mycelium growth of *VdERG2* knockout mutants was sparse and disordered, and the knockout mutant strains significantly reduced the virulence [82]. The number of conidia produced by the *FocSge1* deletion mutant K09 decreased considerably, and no apparent symptoms were observed in bananas inoculated with K09 strains [83]. In the current study, the mycelia of FsGH28c deletion mutant strains were not obviously different from WT strains, but the microconidia showed irregular and hollow shapes. In addition, the FsGH28c deletion mutant strains reduced the ability to utilize different carbon sources. The Δ*FsGH28c* strains significantly reduced the pathogenicity of black pepper.

The yeast signal sequence trap system assay indicated that FsGH28c could be secreted in outer space by *F. solani* to interact with the black pepper host during infection. Furthermore, PnPUB35 was verified to interact with FsGH28c by Y2H and BiFC. Many candidate genes from host plants have been identified to regulate resistance against *F. solani* infection. In *Panax notoginseng* (Burk) F.H. Chen, overexpression of *PnWRKY9* increased the tobacco resistance to *F. solani*, whereas the *PnWRKY9*-RNAi *P. notoginseng* plants increased the susceptibility to *F. solani* [84]. *MdTyDC* and *MdWRKY40* overexpression enhances apple resistance to apple replant disease, which is caused by *F. solani* [85,86]. In cucumber, transgenic overexpressing *CsMYB60* seedlings were more resistant to *F. solani* than the wild-type seedlings [87]. In our research, *PnPUB35*-silenced black pepper plants showed more severe symptoms of *Fusarium* wilt than WT plants, and *PnPUB35* overexpression enhanced *A. thaliana* resistance to *F. solani*. These results indicated that *PnPUB35* positively regulates the black pepper resistance to *F. solani*. In contrast, GhPUB17 is a negative regulator of cotton resistance to *V. dahliae* [35]. In a previous study, PUB17 was proven to positively regulate the resistance of plants to specific pathogens or their effectors through the HR-PCD pathway [88,89]. In addition, PUB17 is localized in the nucleus, but GhPUB17 interacts with GhCyP3 at the plasma membrane and in the nucleus. These indicated that PUBs induced different immune pathways in host plants under different pathogen infections, respectively. However, *F. solani* is a soilborne fungal pathogen and is phylogenetically categorized in the *F. solani* species complex (FSSC) group [59]. Its infection process is different from that of other pathogens, such as *Pst* DC3000 and *P. infestans* [60]. Moreover, PnPUB35 was localized in the nucleus and interacted with FsGH28c at the plasma membrane. Under exogenous application of stress, proteins could change localization in the cell. Like the PHORI in potatoes, its localization was altered from the cytosol to the nucleus in the presence of GA [90]. When *F. solani* WN-1 infected the plant, PnPUB35 was observed at the plasma membrane and in the nucleus (Figure S7) and interacted with FsGH28c to regulate the responses to *F. solani* infection. These demonstrated that plant PUBs act as a sophisticated factor in response to pathogen threats. In this study, the downstream signaling pathway of PnPUB35 in black pepper defense against *F. solani* remains to be explored.

## 5. Conclusions

**Figure 8:**
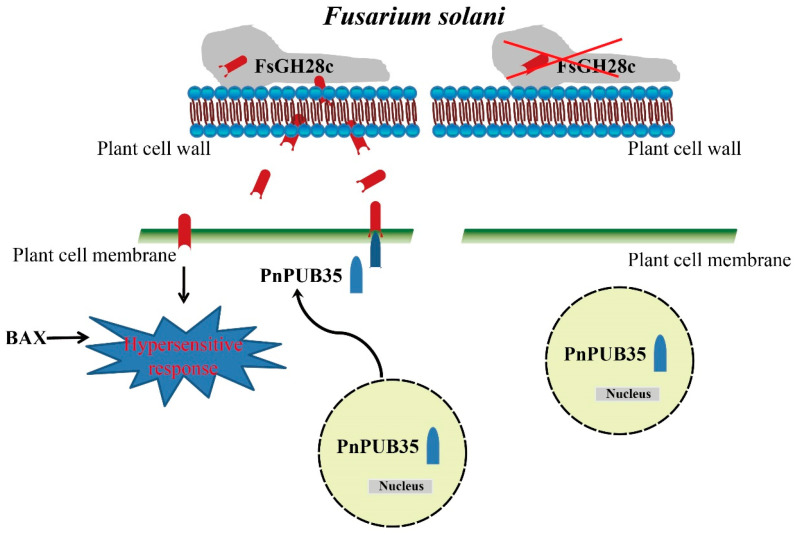
Schematic model of the interaction between FsGH28c and PnPUB35. When *Fusarium solani* infects plants, *F. solani* secretes a virulence factor FsGH28c. FsGH28c can induce immune responses. It is a positive regulator of *F. solani* virulence. The PnPUB35 transfers localization from the nucleus to the plant cell membrane in the presence of *F. solani*, and interacts with FsGH28c to protect the plant against *F. solani*. The red X represents the *F. solani* strain without FsGH28c.

In conclusion, our study demonstrates that FsGH28c, a member of the GH28 family in *F. solani*, functions as a virulence factor, eliciting immune responses in the host. It appears to modulate *F. solani’*s capacity to utilize carbon sources in the soil, and to influence spore development on the host’s root surface during infection. Furthermore, FsGH28c interacts with the U-box protein PnPUB35, which is known to enhance the resistance of black pepper against *F. solani* infection. Our findings suggest that FsGH28c not only triggers a hypersensitive response but also disrupts the interaction with PnPUB35, thereby modulating the host’s resistance to *F. solani*, as illustrated in Figure 8.

## Data Availability

The data that support the findings of this article are available from the corresponding author upon request.

## Supplementary Materials

The following supporting information can be downloaded at: https://www.mdpi.com/article/10.3390/ijms26094189/s1.
