# Supplementary figures and images for: Interaction Between Glycoside Hydrolase FsGH28c from Fusarium solani and PnPUB35 Confers Resistance in Piper nigrum
Source: Int J Mol Sci. 2025 Apr 28;26(9):4189. doi: 10.3390/ijms26094189 (PMC12071851; doi:10.3390/ijms26094189)

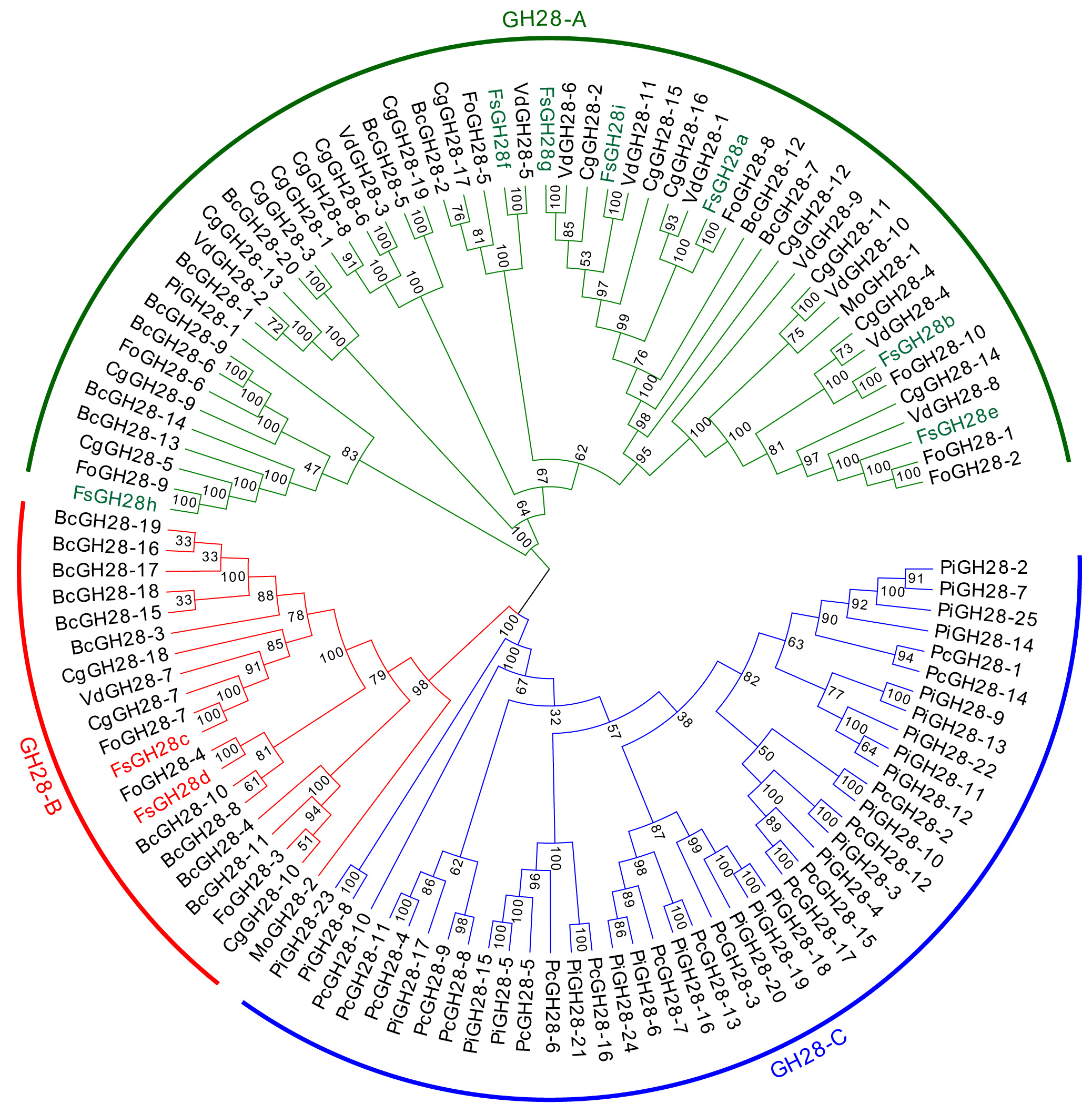

Supplement: Supplementary file 1 [file ijms-26-04189-s001.zip › figure S1.jpg]

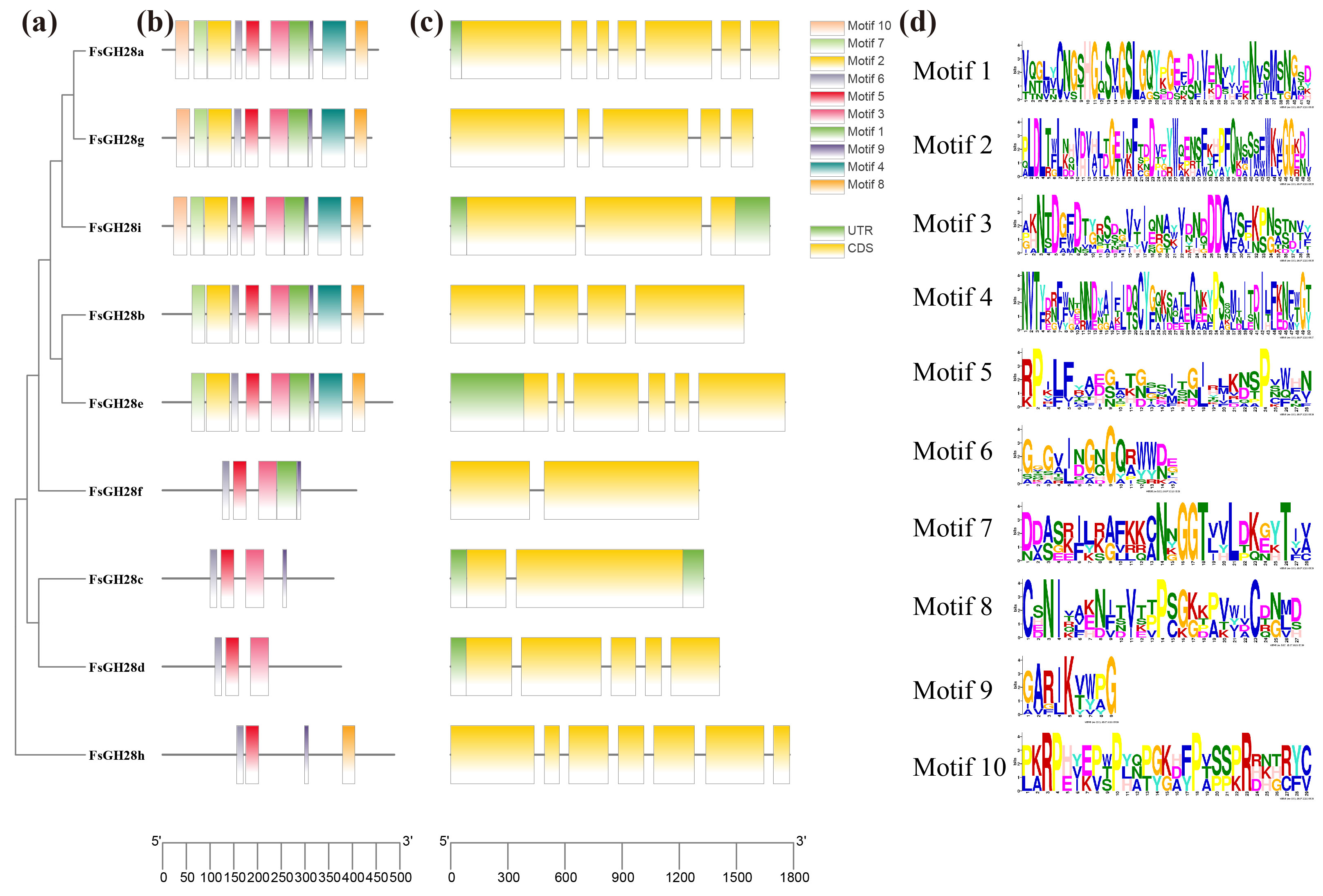

Supplement: Supplementary file 1 [file ijms-26-04189-s001.zip › figure S2.jpg]

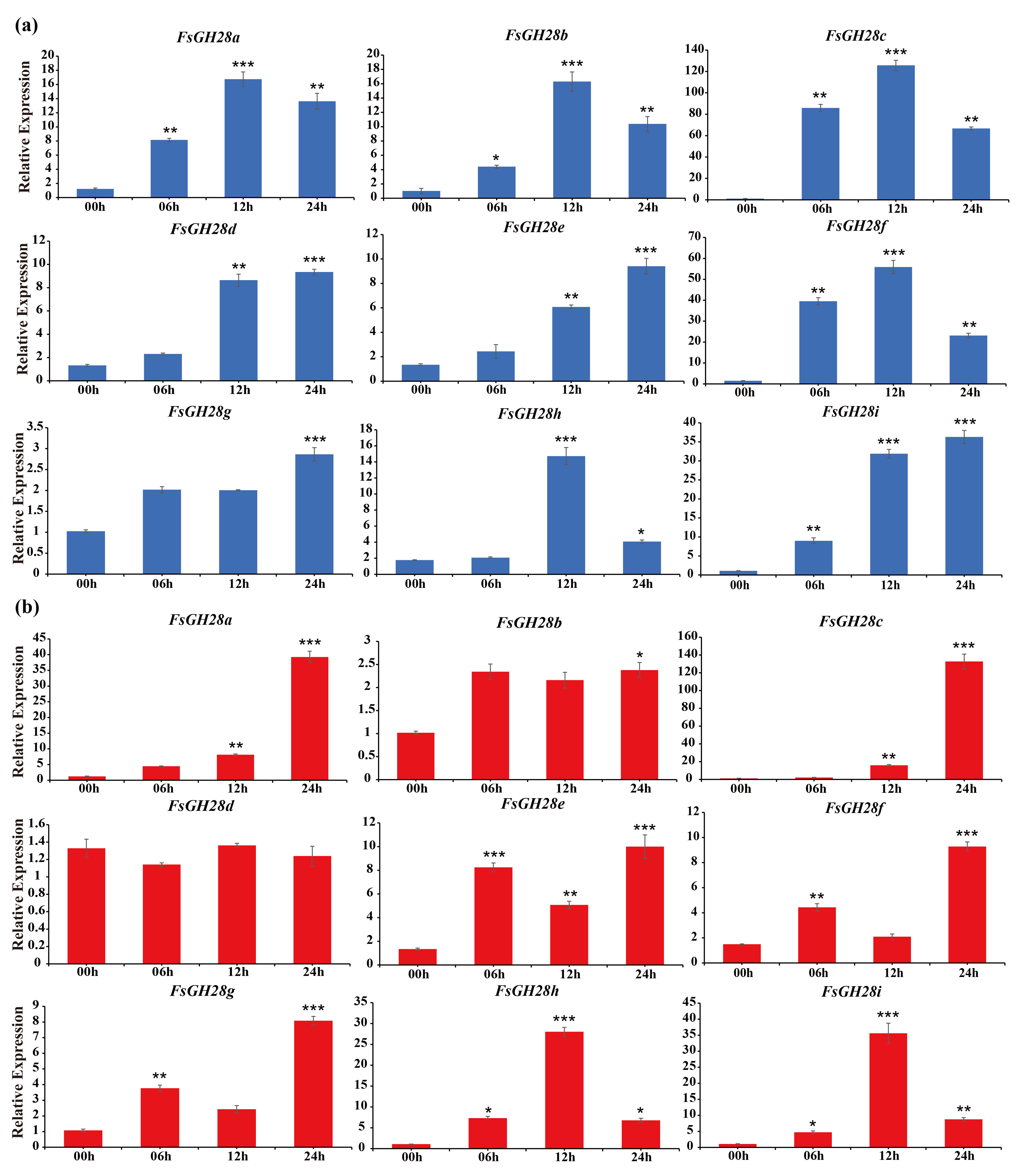

Supplement: Supplementary file 1 [file ijms-26-04189-s001.zip › figure S3.jpg]

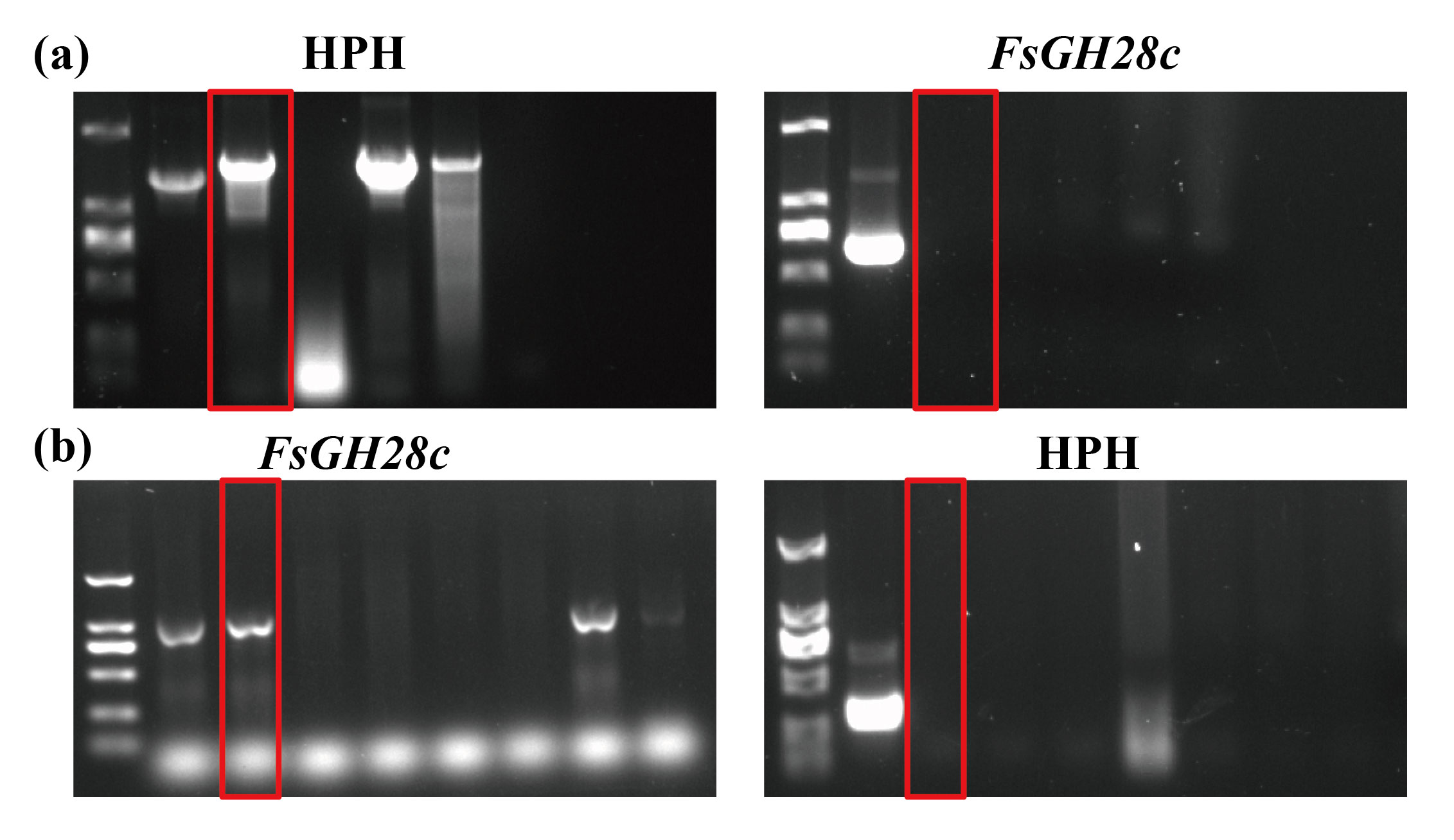

Supplement: Supplementary file 1 [file ijms-26-04189-s001.zip › figure S4.jpg]

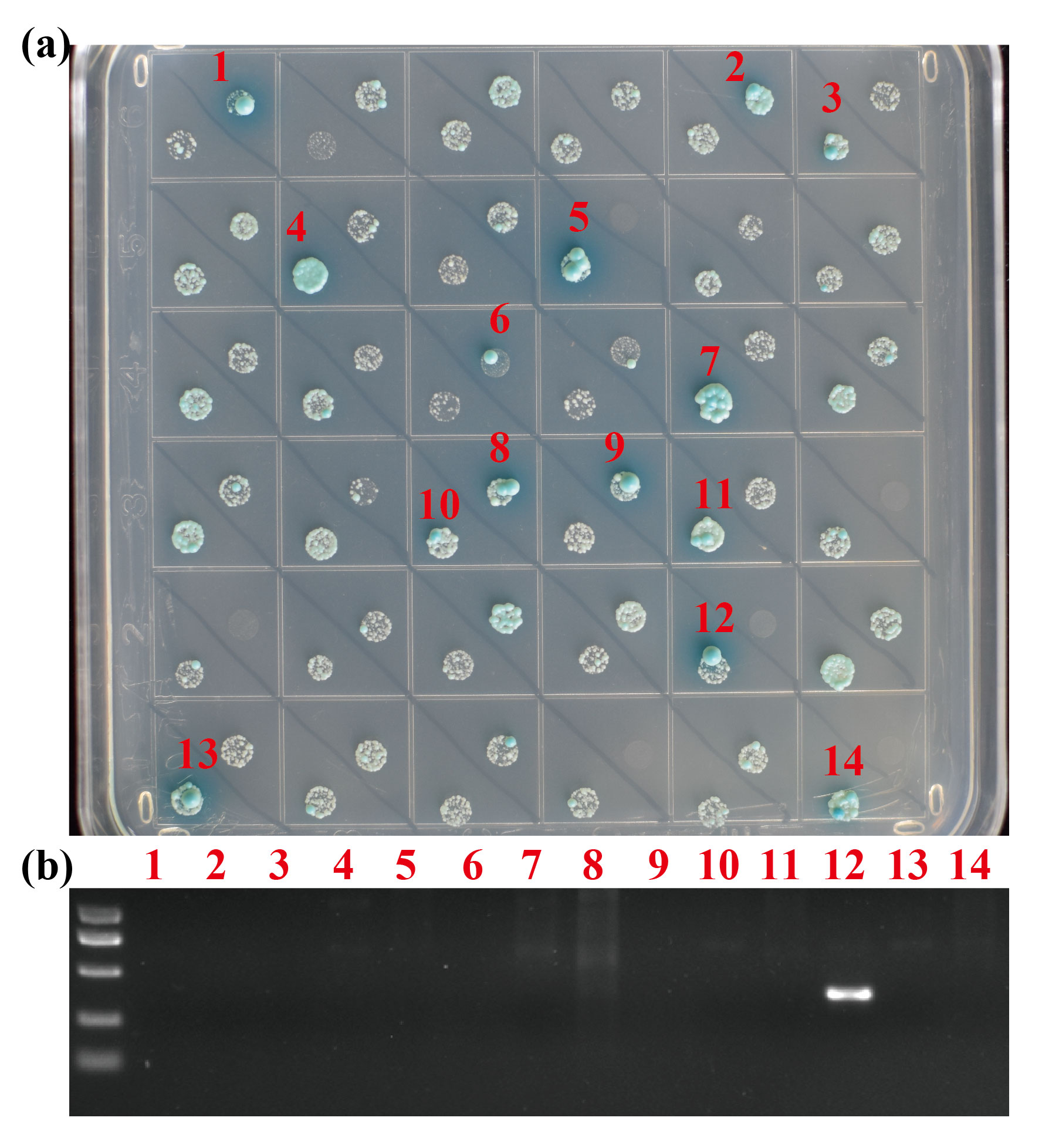

Supplement: Supplementary file 1 [file ijms-26-04189-s001.zip › figure S5.jpg]

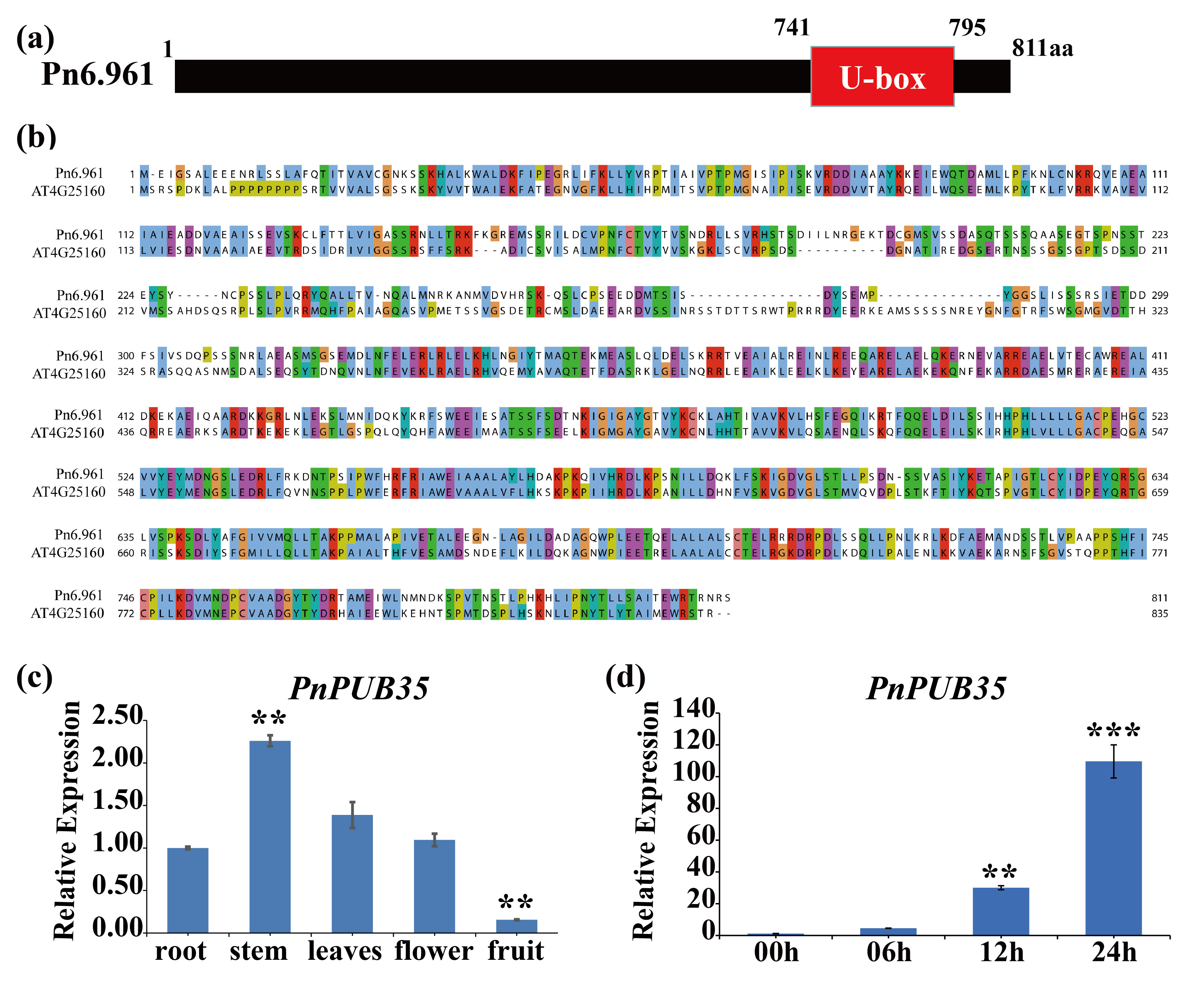

Supplement: Supplementary file 1 [file ijms-26-04189-s001.zip › figure S6.jpg]

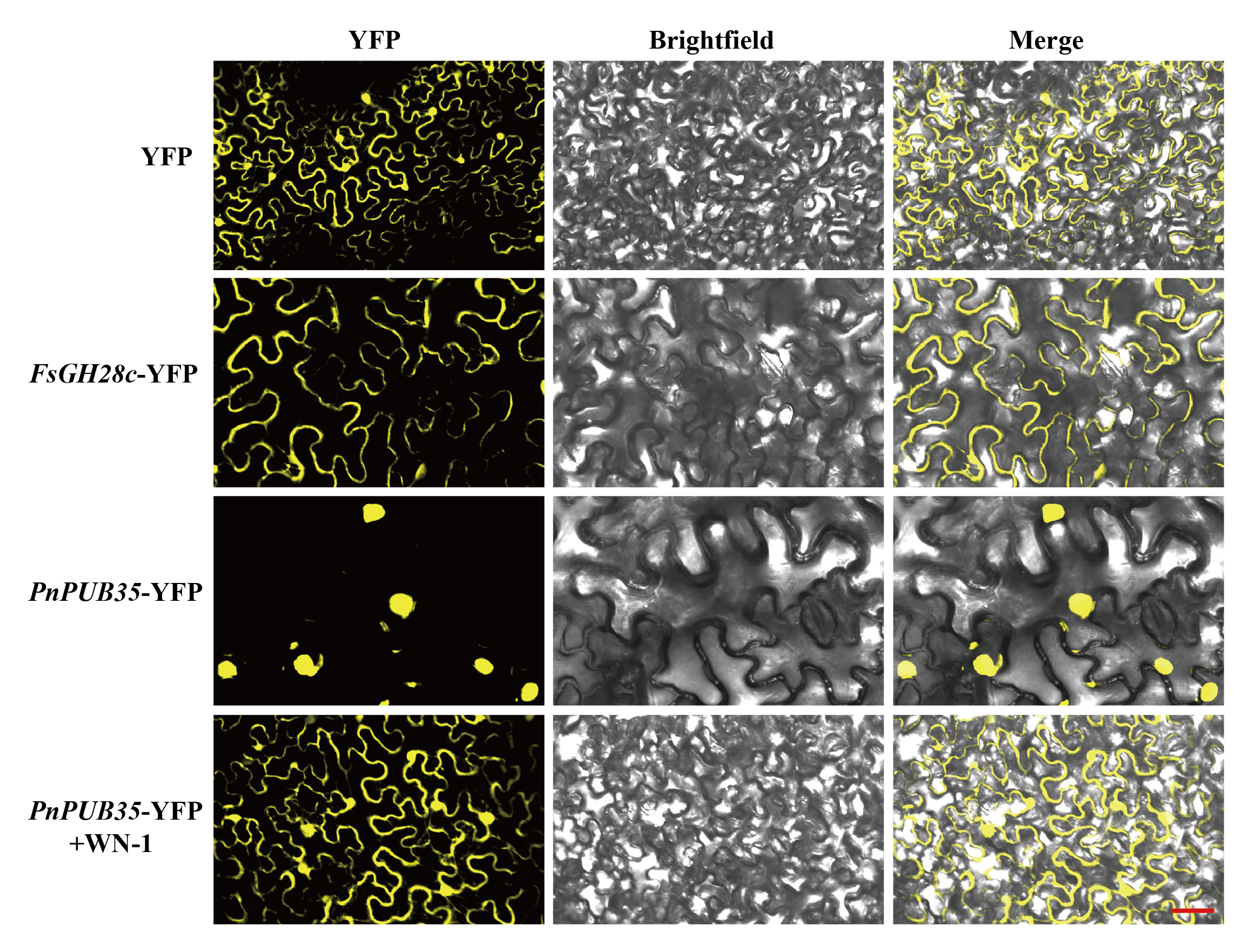

Supplement: Supplementary file 1 [file ijms-26-04189-s001.zip › figure S7.jpg]

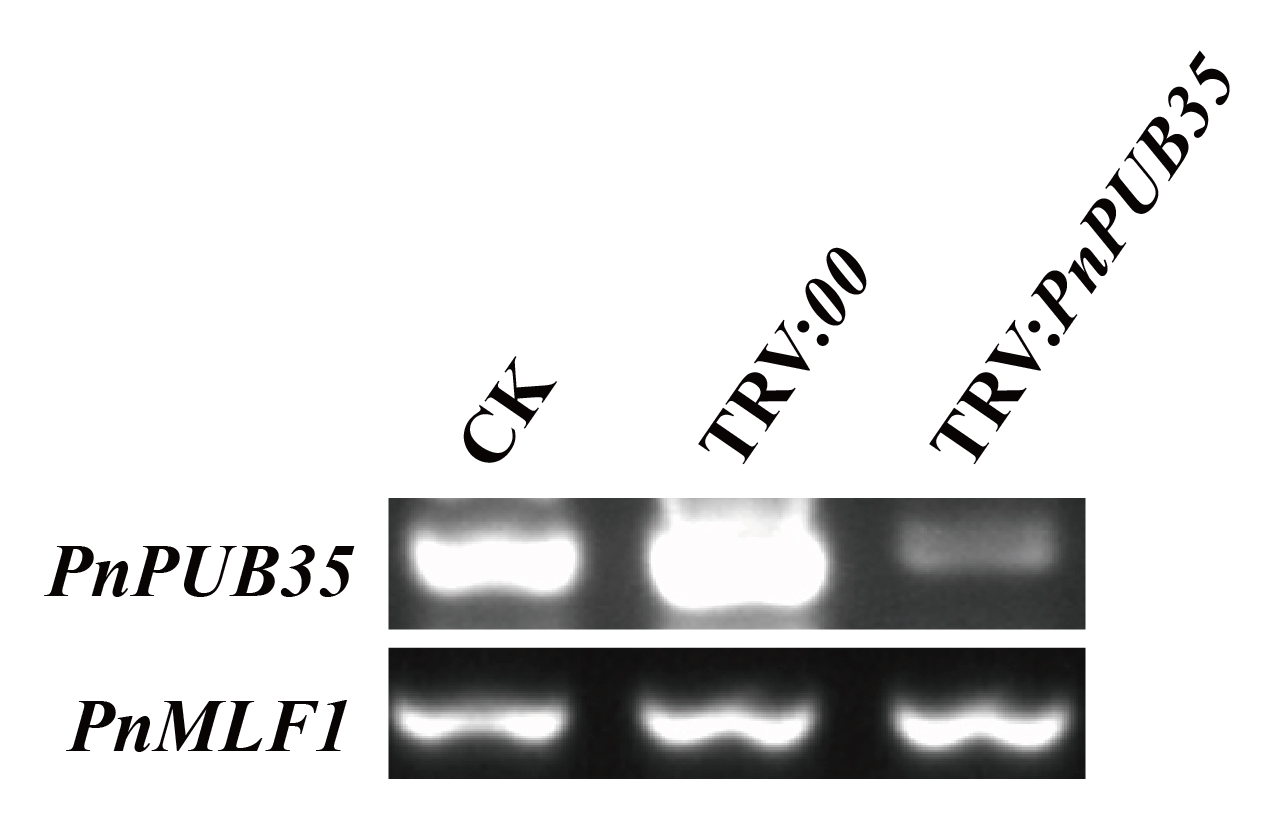

Supplement: Supplementary file 1 [file ijms-26-04189-s001.zip › figure S8.jpg]
